# Unethical Leadership and Employee Extra-Role Behavior in Information Technology Sector: A Moderated Mediation Analysis

**DOI:** 10.3389/fpsyg.2021.708016

**Published:** 2021-10-11

**Authors:** Fengjiao Zheng, Naseer Abbas Khan, Muhammad Waseem Abbas Khan

**Affiliations:** ^1^Center for Environment and Sustainability, University of Surrey, Guildford, United Kingdom; ^2^Department of Industrial Economics and Project Management, South Ural State University, Chelyabinsk, Russia; ^3^Department of Medicine, Rawalpindi Medical University, Rawalpindi , Pakistan

**Keywords:** extra-role behavior, COVID-19, unethical leadership, psychological empowerment, perceived social support

## Abstract

During the COVID-19 pandemic, enterprises were obliged to employ social media and digital tools to complete ordinary work. The pandemic has created a series of complexities and challenges, which have hampered harmonic contact between leaders and followers. The indirect relationship between unethical leadership and extra-role behavior (EXB) *via* psychological empowerment (PYE) is investigated in this study. We also look into the role of perceived organizational support (POS) as a moderator in the link between unethical leadership and PYE, as well as the indirect link between unethical leadership and EXB. Data were obtained from 258 supervisor–employee dyads from various small- and mid-sized information technology (IT) enterprises using time lag data. Unethical leadership has an impact on employee psychological empowerment as well as EXB. The findings of this study indicated that POS also mitigated the negative consequences of unethical leadership on employee psychological empowerment. Similarly, the role of psychological empowerment as a mediator in the link between unethical leadership and employee EXB is influenced by POS. This study will also benefit researchers and practitioners interested in human resource practices in the IT industry.

## Introduction

Management is compelled to develop strategically adaptable enterprises in response to more competitive marketplaces. Fortunately, a new wave of information and telecommunications technologies has set the basis for previously unthinkable new organizational forms (Khan et al., 2020a). The COVID 19 pandemic has forced enterprises to adopt digital working patterns, and the difficult and complicated nature of digital work is raising ethical challenges in the workplace (Weberg and Fuller, 2019). Extra-role behavior (EXB) can also improve an organization’s efficiency and effectiveness by transforming organizational resources, reforming resources, and adapting to changing situations (Labrague, 2020; Khan et al., 2020b). In previous studies on the information technology (IT) sector, EXB by IT company employees has been given a lot of weight. Employee behavior has been found to have a direct impact on their willingness and desire to learn, thereby boosting the institution’s status and distinguishing it from the competitors (Vigoda-Gadot, 2007; Khan et al., 2020c). Extra role play might be advantageous to the enterprise because the IT sector is a spontaneous and humanistic profession that requires working with and managing a variety of technological facilities (Belogolovsky and Somech, 2010). Leaders who do not encourage EXB are more likely to have lower employee achievement and effectiveness (Srivastava, 2017; Khan and Khan, 2021).

Despite the fact that the antecedents of EXB have attracted little empirical attention, scholars have claimed that leadership behaviors play a role in supporting or inhibiting employee performance (Khan, 2021). Cortina (2008) argued that employees expect leaders to pick up on clues about what constitutes appropriate EXB since they set the tone for the entire enterprise. Previous research has shown that leaders’ behaviors have a significant impact (Behery et al., 2012). There has been a growing trend in leadership literature to address the dark and peculiar negative aspects of leadership behavior, as well as their impact on followers (Khoo and Burch, 2008). Unfortunately, unethical behavior by leaders has spread to all types of organizations, both public and private (Mathieu et al., 2014). An increasing number of leadership and management studies are focusing on the negative aspects of leadership, such as disruptive leadership (Goldman, 2009; Bhandarker and Rai, 2019), abusive leaders (Tepper, 2000), and tyrannical leaders (Allio, 2007). Leader behaviors or acts that are illegal or contradict current moral standards are referred to as unethical leadership (Brown and Mitchell, 2010).

Leadership research may aid in the investigation of a leader’s dark side in order to determine its impact on organizations as a result of potential behavioral deviations in employee work performance (Khoo and Burch, 2008). Unethical leaders tries to please senior managers and earn personal favor, but they overlook subordinates’ vital EXB, harming the environment, human resources, and organizational culture in the process (Boddy and Croft, 2016). Similarly, Gan et al. (2019) asserted that unethical leadership jeopardizes individuals’ psychological well-being and that as a result, unethical leadership undermines organizational success.

The purpose of the study is to learn more about how unethical leaders affect employees’ EXB in the IT industry. EXB is vital to remember in the Chinese context since most Chinese workers have a high power distance orientation, which suppresses the EXB of employees who are focused on work-related matters (Liu et al., 2010; Zhang et al., 2011). Extra-role behaviors are defined as those that benefit or intend to benefit the company and are directly linked to the role and expectations. In the workplace, this is referred regarded as organizational citizenship behaviors (Zhu, 2013). The extra-role of workers supports industry-related behavior. Employees’ EXB has been linked to positive outcomes (like personal job performance and organizational effectiveness; Ng and Feldman, 2012; Frazier and Bowler, 2015). Given the potential benefits of EXB, researchers focused on the antecedents of EXB that support optimal practices.

To further understand why unethical leadership is linked to EXB, this study investigated the mediating effect of psychological empowerment (PYE) or the assumption that showing unethical leadership will negatively affect employee EXB. Although studies have linked unethical leadership with work behaviors using job satisfaction, strain, and commitment (Weberg and Fuller, 2019), such mediating mechanisms cannot completely explain the effect of toxic leadership on EXB. Extra-role voice behavior, unlike other forms of employee behavior that promote cooperation, can produce organizational disruption and high individual costs for performers, making employees apprehensive of engaging in it (Turnley and Feldman, 1999).

**Figure 1:**
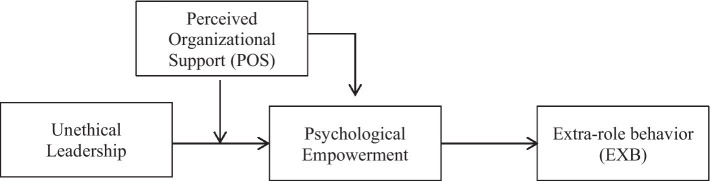
Research Model.

According to Turnley and Feldman (1999), the key to fostering EXB is to influence employees’ expectations of empowerment, which are not reflected by strain, happiness, or dedication. Employees who are at ease with interpersonally hostile acts are more likely to engage in constructive behavior (Valentine and Edmondson, 2014). The PYE paradigm is easily relevant to unethical leadership effects on extra-role activities as a result of such an affect-laden cognitive perspective (Van Dyne and LePine, 1998; Khan et al., 2021). Furthermore, the unethical leadership paradigm suggests that employees might use a shield to protect themselves from the negative impacts of unethical leadership; as a result, unethical leadership does not affect all employees equally (Ferris et al., 2002). We question if unethical leadership has a synchronized influence on psychological empowerment and EXB for which employees. Employees who become victims of unethical leadership activities may benefit from perceived organizational support (POS). Employee perceptions of how much the organization values their contribution and cares about their well-being are referred to as POS (Eisenberger et al., 1986). According to a previous study, POS has a significant impact on employee performance and well-being. POS can mitigate the negative effects of unethical leadership on psychological empowerment and EXB (Eisenberger et al., 1990). We aimed to provide a moderated mediation model that takes into account why unethical leadership is associated with EXB by looking at the intervening role of psychological empowerment, as well as how the relationship proceeds by looking at the POS boundary condition (see Figure 1).

## Theory and Hypothesis

### Unethical Leadership, Psychological Empowerment, and EXB

Unethical leader behavior is currently receiving a lot of attention in the media and in the business literature because of the prevalence of many types of unethical behavior in the workplace as a result of leaders’ corruption or failure to follow moral norms (Gan et al., 2019). Unethical leadership has an impact on employee psychological outcomes, and employee psychological empowerment helps employees cope with the negative consequences of unethical behavior (Kalshoven et al., 2016). Because workers in highly unethical organizations are unable to get crucial indicators for understanding behaviors and prospective outcomes, the behavior–outcome relationship tends to be a “black box” defined by incompatibility (Khan et al., 2020d). Employees are less likely to notice useful cues that could aid them in deciphering the intricacies. Employees disregard their ability to predict future outcomes, viewing the situation as a negative and unmanageable threat (Weberg and Fuller, 2019). When confronted with this difficulty, employees may see extra-role performance as dangerous. According to one study, having limited access to corporate culture information increases sensitivity and defensiveness (Edmondson, 1999). Several studies have indicated that ethical leadership promotes psychological empowerment (Dust et al., 2018; Suifan et al., 2020). According to a meta-analytic review, unethical leadership induces psychological stress (Matos et al., 2018). Thus, we assume that:

*H1*: Unethical leadership has a negative impact on psychological empowerment.

### Psychological Empowerment as a Mediator

Psychological empowerment has been shown to be a powerful motivator that can improve employees’ work engagement and extra-role performance (Ugwu et al., 2014; Bano et al., 2019). Psychological empowerment has also been shown to have a beneficial impact on job satisfaction (Amundsen and Martinsen, 2015) and is a strong predictor of work engagement (Dewettinck and Van Ameijde, 2011). According to social exchange theory, the nature of the individual–organization association has a profound influence on human behavior (Blau, 1964). When employees believe their workplaces are psychologically safe, they are more likely to see their association with the employer as a relational rather than an economic one, reciprocating by expressing voluntary tasks in the organization. This indicates a constructive association between psychological empowerment and employee behavior related to performing extra-role. To better understand the dynamics of unethical leadership impact, researchers have long proposed that the unethical leadership–outcome nexus be analyzed in its entirety, including mediating effects (Khan et al., 2020e). Therefore, we examine the mediating role of psychological empowerment in advancing a system that connects unethical leadership and EXB. Employees must consider organizational climate features when determining whether or not to partake in employee behavior (Tangirala and Ramanujam, 2008).

People who work in extremely hazardous environments are more likely to dwell on the negative and uncontrollable aspects of a crisis (Ferris et al., 2002), and they have even less psychological empowerment. Thus, in a less psychologically safe business environment, people are more likely to engage in activities relating to resource conservation and strict regulation systems, which are linked to activity limitation and revealed in the status quo (Staw et al., 1981). Employees who have psychological empowerment as a result of an unethical work environment, on the other hand, are less likely to challenge the organization because insufficient psychological empowerment is a concern for them (Webster et al., 2016). Hence, unethical leadership becomes a significant impediment to IT industry staff using psychological empowerment to express their experiences and other voluntary actions at work. The evidence for psychological empowerment mediating effect has been increasing. For example, psychological empowerment was established to mediate the effects of change-oriented leadership (Detert and Burris, 2007) and ethical leadership (Walumbwa and Schaubroeck, 2009) on follower EXB. Several researchers have focused on positive leadership, but none have looked at the effect of an unethical workplace environment on psychological empowerment and employee EXB in the IT industry field. Unethical leadership could be the most potentially applicable construct for relating an unethical work atmosphere to decreased psychological empowerment and EXB. Hence, we propose:

*H2*: Psychological empowerment mediates the association between unethical leadership and EXB.

### Moderating Influence of POS

Organizational Support Theory argues that individuals tend to personify their organization by considering it as a personality with either good or malignant intents toward them (Eisenberger et al., 1986, 1990). The positive effects of POS on outcomes that benefit both individuals and enterprises are explained by two basic factors. On the one hand, the Social Exchange Theory (Blau, 1964), based on the reciprocity norm (Gouldner, 1960), proposes that employees who feel supported by their organization will pay back their obligation and repay the firm’s caring.

We propose that POS can act as a buffer against the negative effects of unethical leadership. Companies can increase workers’ views of authority while also lowering the negative impacts of confusion that come with unethical leadership (Miller et al., 2008; Khan et al., 2020f). Employees’ perceptions of organizational support might help them recognize decision rules and retain additional resources, reducing the ambiguity that comes with challenging situations (Li et al., 2014). Employee morale and EXB are lowered to a minimum as a result of unethical leadership (Wang et al., 2015). Organizations use rewards to communicate to specific employees that they have received organizational support, which helps to foster workforce identification (Li et al., 2014). People who believe their organization is on their side are more likely to trust authorities and take control over their surroundings (Khan and Khan, 2019; Khan et al., 2020g). By establishing a strong relationship with the organization as a shield (Stamper and Masterson, 2002), which acts as a shield by defending employees’ higher EXB interests, the organization will gain more employee support in adverse situations. Research backs up the premise that social support and confidence in coworkers buffer the detrimental impacts of unethical leadership on job outcomes (Vigoda-Gadot and Talmud, 2010). To summarize, when individuals identify as organizational support receivers, they are less likely to perceive unethical leadership as a less threat, reducing the negative impact of unethical leadership on psychological empowerment and EXB. Thus, this study proposes:

*H3*: POS moderates the negative association between unethical leadership and psychological empowerment in this way that high POS weakens rather than strengthens this association.

The aforementioned statements are part of a larger context in which psychological empowerment mediates both unethical leadership and psychological empowerment, as well as unethical leadership and EXB, and POS moderates both unethical leadership and psychological empowerment. POS may potentially moderate the efficacy of the mediating mechanism for psychological empowerment in the association between unethical leadership and EXB – a moderated mediation model (Edwards and Lambert, 2007). Organizational support is more likely to provide proper knowledge and social reinforcement, which can lead to psychological empowerment and, ultimately, EXB, so POS is critical for lowering the indirect effect of unethical leadership on EXB through psychological empowerment. Thus, we suppose as:

*H4*: The mediating impact of psychological empowerment on the unethical leadership EXB association is moderated by POS, with the mediating effect lower when POS is high rather than low.

## Materials and Methods

### Sample and Procedures

We gathered data from supervisor–employee dyads from several small- and mid-sized IT enterprises in China’s Anhui and Jiangsu provinces for this study. Employee EXB is important in IT enterprises because they want their employees to create voluntary interests in order to accomplish extra tasks that help enhance quality and service processes (Turnley and Feldman, 1999). The most significant feature of this study is that data were collected in two-time waves employing a time lag approach. In a time-lag survey, data were collected at multiple intervals, reducing the possibility of common method biases (Podsakoff et al., 2003) while simultaneously giving respondents ample time to observe and respond to the questionnaire (Detert and Burris, 2007). We developed an online and offline survey questionnaire in English, which was subsequently translated into Chinese using a process called translation back-translation (Brislin, 1980). Student volunteers were engaged to collect data through social media and email. In addition, offline survey questionnaires were mailed to the Human Resource Departments of the respective IT enterprises. As part of the data gathering procedure, 206 randomly selected employees were given online and offline surveys and asked to nominate their immediate superiors. The survey was designed using a five-point Likert scale ranging from 1 to 5, with 1 indicating strongly disagree and 5 indicating strongly agree. In the first wave, we requested permission from 412 matched employees and their immediate managers to participate in the survey, and we received 312 responses. Employees provided demographic data as well as information on unethical leadership and POS. In the second wave, psychological empowerment data were collected from employees, while employee EXB data were collected from immediate supervisors. Some questionnaires were incomplete or incorrectly rated, and these were excluded from the final sample for analysis. Thus, there were a total of 258 final responses received. Table 1 shows the demographics of the respondents.

**Table 1:** Demographics.

Variables	*N*	Percentage
Gender		
Female	109	42.25
Male	149	57.75
Age		
Up to 24years	103	39.92
25–30years	94	36.43
31–35years	37	14.34
36–40years	11	02.84
Above 40years	13	03.55
Qualification		
Undergraduate	48	18.60
Graduate	122	47.29
Masters/PhD	88	34.11
Experience		
Up to 05years	102	39.53
6–10years	106	41.09
11–15years	32	12.40
Above 16years	18	06.98

*N*=258.

### Measurement Scale

The unethical leadership scale was developed by Pitesa and Thau (2013) and used by Gan et al. (2019). Employees were asked to rate their degree of agreement on a five-point Likert scale (1=strongly disagree, 5=strongly agree). Sample item included: “*My supervisor never discussed confidential company information with an unauthorized person*.” To assess POS, we used the scale developed by Eisenberger et al. (1986). The response options ranged from 1 to 5, with 1 representing “strongly disagree” and 5 representing “strongly agree.” A sample item was “*organization really cares about my well-being*.” To assess psychological empowerment, we used a scale developed by Spreitzer (1995). The response options ranged from 1 to 5, with 1 “strongly disagree” and 5 “strongly agree.” A sample item was “*I can decide on my own how to go about doing my work*.” We used a five items measurement scale of EXB adapted by Bettencourt and Brown (1997). Sample item includes “*This employee helps customers with problems beyond what is expected or required*.” All of the items were rated on a five-point Likert scale ranging from 1 (strongly disagree) to 5 (strongly agree).

### Data Analysis

To ensure the validity of the proposed study variables, we used AMOS version 24.0 for CFA testing in this study (see Table 2).

**Table 2:** Correlation matrix.

	EXB	POS	Unethical leadership	Psychological empowerment
EXB				
POS	0.43^**^	1		
Unethical leadership	−0.38^**^	−0.15^**^	1	
Psychological empowerment	0.37^**^	0.18^**^	−0.45^**^	1

** *p*<0.01;

* *p*<0.05 (two-tailed).

The items in each construct with the highest and lowest factor loads are merged first, followed by the items with the highest and lowest factor loads, and finally, all items are assigned to one of the indicators depending on the factor analysis results. In this study, four variables were used to assess the CFA model: unethical leadership, employee psychological empowerment, POS, and employee EXB. In this study, the tucker-lewis index (TLI), comparative fit index (CFI), and root-mean-square error of approximation (RMSEA) tests were used to evaluate model fit. The model fit was found to be satisfactory: *χ*^2^=658.965; df=392; CFI=0.982, TLI=0.984; RMSEA=0.043 (see Table 3).

**Table 3:** Results of confirmatory factor analyses.

Models	*χ* ^2^	df	TLI	CFI	RMSEA
Four factors model	658.965	392	0.984	0.982	0.043
Three factors model UL, and PYE combined	863.291	402	0.961	0.963	0.067
Two factors model UL, POS, and PYE combined	1,098.112	413	0.956	0.962	0.068
Single factor model	1,129.824	417	0.957	0.957	0.069

UL, unethical leadership; PYE, psychological empowerment; POS, perceived organizational support; TLI, tucker-lewis index; CFI, comparative fit index; and RMSEA, root-mean-square error of approximation.

After validating convergent validity, the data show that all variables loading have a significant impact on latent constructs. The discriminant validity of the four proposed constructs was then examined using several models on the four-factor model used in the study. The results of the fit index imply that the data better fit the impact of the four-factor model (see Table 2). As a result, the study’s key constructs’ outcomes are more distinct and inclusive of the findings. Based on the findings, four constructs were analyzed further.

## Results

Using hierarchical multiple regression analysis, the hypotheses provided in this study were tested. Hypothesis H1 expected a link between unethical leadership and psychological empowerment among employees. Employee psychological empowerment was found to be significantly negatively related to unethical leadership (*r*=−0.41, *p*=001), supporting H1 (see Table 4).

**Table 4:** Mediating role of PYE and moderating role of POS.

	Dependent variable					
Predictors	Psychological empowerment			EXB		
	M1	M2	M3	M4	M5	M6
Unethical leadership	−0.41^***^	−0.39^***^	−0.32^***^	−0.21^***^		−0.06
Perceived organizational support		0.30^*^	0.34^*^			
POS×UL			0.21^**^			
Psychological empowerment					0.31^***^	0.28^***^
*R* ^2^	0.32^***^	0.33^***^	0.34^**^	0.06^**^	0.10^***^	0.11^***^
Δ*R*^2^	0.32^***^	0.01^*^	0.01^**^	0.06^**^	0.11^***^	0.06^***^
*F*	38.11^***^	4.48^*^	11.75^**^	4.70^**^	9.90^***^	26.13^***^

* *p*<0.05;

** *p*<0.01;

*** *p*<0.001.

The current study used Baron and Kenny (1986) approach to evaluate mediating effects. Four conditions must be met for this approach to consider the mediation effect. to investigate mediating effects. First, there should be a significant relationship between independent variables and mediators. Second, there should be a significant link between the independent and dependent variables. Third, there should be a significant link between the mediator and the dependent variable. Fourth, the independent and dependent variables should have an insignificant link in the presence of a mediator.

Table 3 shows a significant relationship between unethical leadership and psychological empowerment (*r*=−41, *p*=001), showing that the first criterion was met. The results (see Table 3) showed a significant link between unethical leadership and employee EXB (*r*=−0.21, *p*=0.01), demonstrating that the second condition is satisfied. Similarly, the results (Table 3) indicate a significant relationship between psychological empowerment and EXB (*r*=0.31, *p*=001), which meets the third condition. Furthermore, the findings of the study (see Table 3) show that in the presence of psychological empowerment, the link between unethical leadership and Employee EXB is not significant (*r*=−0.06, ns), indicating that the fourth condition is satisfied. Employee psychological empowerment was found to play a significant role in moderating the link between unethical leadership and employee EXB, indicating that H2 was supported.

According to H3, POS is expected to moderate the link between unethical leadership and psychological empowerment. H3 was supported by the finding that the interaction between unethical leadership and POS was significantly linked with psychological empowerment (*r*=0.21, *p*=01).

**Figure 2:**
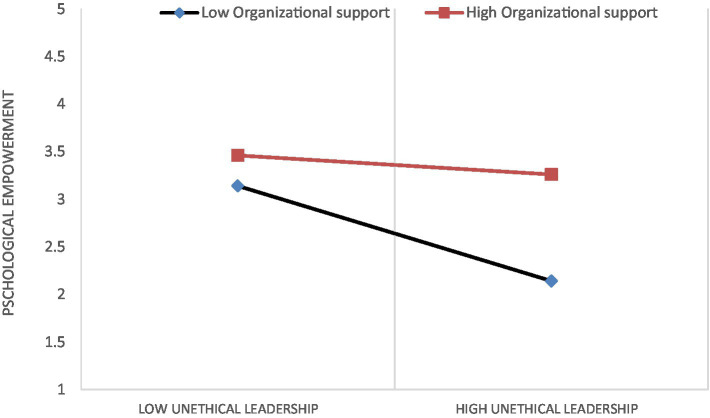
Moderating role of perceived organizational support in the relationship between unethical leadership and psychological empowerment.

Aiken and West (1991) approach was used to map the interactive effect of POS to evaluate if it had a moderating effect (Aiken and West, 1991). Figure 2 depicts interaction patterns that are consistent with H3 predictions. There was a negative correlation between unethical leadership and psychological empowerment when the POS was low (*r*=−0.31, *p*=001), but when the POS was strong (*r*=0.40, *p*=001), the association became significant and positive (*r*=0.40, *p*=001).

Preacher et al. (2007) constructed a process macro that ran a bootstrapping test to determine the indirect impacts of mediation on different levels of moderators. Because it uses bootstrapping to moderate the normality of the mediation effect distribution, the CI is distinguished from other conventional methods (Preacher et al., 2007). The bootstrapping method evaluates the effect of the generated data set by replacing the original data set with an observational estimate of the numbers. The direct effect of the predictor on the independent variables is obtained using this method. It also has an indirect impact on the outcome variable, which is passed through to the mediator (psychological empowerment; MacKinnon et al., 2004). The 95 percent CIs for indirect effects *via* POS were created using Process Model 7 and 5,000 bootstrapping samples.

Table 5 shows the CIs for the bootstrapping test on three POS values: one SD below the mean, one SD for the mean, and one SD above the mean. If there are values between the low and high CIs that are not zero, the CIs are statistically significant (Hayes, 2013). Table 5 illustrates the bootstrapping CIs for indirect psychological empowerment influence, with POS values ranging from one SD above mean (−0.18 to −0.05), mean (−0.20 to −0.11), and one SD below mean (−0.26 to −0.12). There is a significant indirect mediation effect of unethical leadership on employee EXB *via* psychological empowerment because this does not include zero. Under the conditions of one SD above, mean, and one SD below, it can find a significant moderated mediation effect. Thus, the H4 was supported.

**Table 5:** Bootstrap results for conditional indirect effects psychological empowerment.

Perceived organizational support	Boot indirect effects	Boot PYE	Boot lower limit 95% CI	Boot upper limit 95% CI
−1 SD	−0.17	0.03	−0.26	−0.12
Mean	−0.11	0.02	−0.20	−0.11
+1 SD	−0.09	0.03	−0.18	−0.05

CI, confidence interval; Bootstrap sample size=5000.

## Discussion

Despite the fact that unethical behavior in corporate culture is a widespread occurrence (Brown and Mitchell, 2010), and most firms understand the relevance of extra-role practices, leadership abuse is considered as a barrier to any society’s and organization’s growth and development. Extra-role behavior is becoming increasingly crucial in the IT business to address the difficulties of digital safety and harmonic online contact between IT employees (Pitafi et al., 2020). However, there are significant gaps in the literature regarding unethical leadership and employee psychological and behavioral outcomes in small- and mid-sized IT enterprises that must be addressed.

It is commonly considered that an unethical work environment may negatively affect employee behavior and diminish employee desires to engage in additional activities that are beneficial to the organization (Tangirala and Ramanujam, 2008). There is a paucity of research linking unethical leadership to employee extra-role actions and behavior, as well as studies examining the boundary conditions of unethical leadership’s effects in the context of the IT industry. This study proposed four hypotheses to analyze the effects of unethical leadership on employee EXB. This finding is in line with previous research, which has shown that ethical leadership has a positive effect on psychological empowerment. As a result, unethical leadership might have a negative impact on psychological empowerment. This study also found that psychological empowerment has a significant mediation influence on the link between unethical leadership and employee EXB, which is in line with previous studies (Dust et al., 2018).

Furthermore, the POS impact was found to have significant in moderated mediation analysis, which is consistent with previous studies (DeConinck, 2010; Khan et al., 2021). Our four hypotheses were all supported in the end. Prior studies in a different field have found consistent outcomes, specifically in the context of ethical leadership. However, this article addresses a gap in the research by proving a link between unethical leadership and employee behavior as well as investigating the boundary conditions of unethical leadership’s consequences, notably in the IT sector. I’ is also consistent with previous practice (Detert and Burris, 2007) to combine mediators and moderators into a single model.

### Theoretical Implications

On a theoretical level, this research contributes to a new understanding in the area of the influence of unethical leadership on employee psychological empowerment and employee EXB in the IT industry. First, the findings support the view that psychological empowerment and EXB are more than just a tool for LMX, job happiness, and positive affectivity, although there is a substantial correlation between psychological empowerment and these three control variables. In the context of a sample collected from China, our proposed study framework was supported. The impact of unethical leadership on employee psychological and behavioral consequences is explored in our model. The findings supported the model’s validity and usefulness in determining the impact of unethical leadership on employee behavior using a moderated mediation model. Employees and leaders in the IT sector participated in this study, and their responses were recorded at various time intervals. The findings add to our knowledge of the factors that influence employee EXB. Several previous studies have backed up our findings, indicating that leadership, as well as psychological and organizational mechanisms, can influence employee extra-role actions and behaviors (Khan et al., 2019, 2020e).

Second, the moderating effects lead to a more nuanced picture of how unethical leadership–psychological empowerment–EXB is affected by POS. We predicted that organizational support buffers the negative effects of unethical leadership. As a result, employees who are supported by their employers are more likely to have interpretative experiences that will guide their future behavior. Employees with POS, on the other hand, are more likely to have a high level of psychological empowerment and to perform extra-role in a highly unethical leadership than non-POS employees. This is one of the first studies in the IT industry to employ the psychological empowerment approach to describe the link between unethical leadership and EXB.

Third, we sought to contribute to research strategy to test by identifying psychological empowerment as a mediator (cognitive mechanism) in the relationship between unethical leadership and EXB. We looked at the surprising effect of psychological empowerment in predicting employee EXB, which has been established as a key psychological variable. Our model better explains how unethical leadership affects employee EXB, as well as who is most affected by unethical leadership in terms of psychological empowerment and EXB, by combining mediation and moderation. Our findings not only back up and explain assertions that the IT industry has a strong link to employee EXB, but they also provide ways to mitigate the harmful consequences of unethical leadership. Finally, in the context of China, this study adds to academics’ understanding of the impact of unethical leadership on employee psychological (psychological empowerment) and behavioral outcomes (EXB). The majority of previous research looked at a related research framework in the setting of western countries.

### Managerial Implications

This study provides several managerial implications that can assist executives, managers, and policymakers in reducing the negative consequences of unethical leadership. First, the outcomes of this study will benefit the IT industry by allowing us to better understand the factors that influence EXB. Workers’ EXB is crucial in making this field environmentally and socially acceptable. IT industries are environmentally concerned. Extra-role performance, according to our findings, is a psychological phenomenon that is influenced by situational factors (e.g., unethical leadership, POS) *via* psychological empowerment. According to the findings of this study, the first step in avoiding unethical leadership behavior can be performed. Second, individuals’ reactions to an unethical organizational atmosphere are influenced by their prior experiences; therefore, enterprises may need to foster a specific environment, consistent procedures, and success in order to promote extra-role activities (Ferris et al., 2002). Employees react to unethical leadership based on their own experiences; thus, organizational leaders and executives should impose ethical norms and standards (Lapalme et al., 2009). Employees may be motivated to perform extra-role activities to increase organizational performance if they work in an ethically comfortable environment with regular procedures and a formal complaint system.

Third, unethical leadership practices can be reduced by managers and immediate supervisors engaging in psychological counseling and mentoring. It is suggested that the IT industry organizes mentorship and training sessions to alleviate stress and psychological pressure on managers. Finally, the IT industry should develop a recruitment strategy that requires all selected applicants to be reviewed on an ethical and good character basis before being considered for managerial positions. In the recruitment process, people with strong ethical values and proactive personality attributes should be given priority. Finally, according to the findings of this study, psychological empowerment and POS are significant predictors and influences of extra-role activities among employees. IT enterprises should take corrective action to encourage psychological empowerment and POS amid unethical leadership. Increased POS has been shown to improve psychological empowerment at higher levels, reducing the negative association between unethical leadership and psychological empowerment. Leadership approaches (such as change-oriented leadership and ethical leadership) have been shown to improve psychological empowerment at higher levels (Detert and Burris, 2007).

### Limitations and Future Research

This study, like all others, has several limitations that should be considered before interpreting the findings. First, the data for this study came from different sources (including leaders and employees) and different time periods; therefore, there is no serious issue of common method bias. The findings of larger firms may differ from ours because the data were acquired from small- and mid-sized IT enterprises. Second, demographics such as gender, education, experience, age, and occupation were included as control variables in this study. Future research can look into the effect of demographics in better understanding the link between unethical leadership and employee EXB. Third, because our findings are based on data obtained just from IT firms in two Chinese provinces, the findings of this study cannot be generalized to other industries or geographical locations. Findings from research undertaken in other regions of the world may differ from ours.

Third, a broad employee EXB measure was used in this study. Employee EXB has been related in a variety of ways, both favorably and adversely, to unethical leadership and psychological empowerment. According to Liang et al. (2012), Psychological empowerment is positively related to both positive and negative EXB temporal variation to the same degree, dispelling concerns that it affects positive and negative EXB differently. More research into the impact of unethical leadership on all aspects of EXB is needed in the future.

Finally, this study used POS and psychological empowerment as a moderator and mediator in the context of a sample obtained from China. China has a high level of collectivism and reliance on established groups. In terms of cultural norms, this culture differs from western democratic societies. Comparing the impact of POS on the link between unethical leadership and employee EXB should be done with caution.

## Conclusion

The current study sheds light on crucial concerns about unethical leadership and EXB, revealing POS as a key contingent factor and psychological empowerment construct for successfully mediating the unethical leadership–EXB relationship. This study has significant organizational implications since it offers numerous strategies for decreasing the negative effects of unethical leadership and encouraging employees to engage in extra-role activities. Furthermore, the findings of this study can be used to begin further research into additional variables and the underlying mechanisms that allow EXB to perform.

## Data Availability Statement

The raw data supporting the conclusions of this article will be made available by the authors, without undue reservation.

## Ethics Statement

The studies involving human participants were reviewed and approved by Departmental Ethics Committee, South Ural State University. The patients/participants provided their written informed consent to participate in this study.

## Author Contributions

All authors listed have made a substantial, direct and intellectual contribution to the work, and approved it for publication.

## Conflict of Interest

The authors declare that the research was conducted in the absence of any commercial or financial relationships that could be construed as a potential conflict of interest.

## Publisher’s Note

All claims expressed in this article are solely those of the authors and do not necessarily represent those of their affiliated organizations, or those of the publisher, the editors and the reviewers. Any product that may be evaluated in this article, or claim that may be made by its manufacturer, is not guaranteed or endorsed by the publisher.
